# Highly Variable Rates of Genome Rearrangements between Hemiascomycetous Yeast Lineages

**DOI:** 10.1371/journal.pgen.0020032

**Published:** 2006-03-10

**Authors:** Gilles Fischer, Eduardo P. C Rocha, Frédéric Brunet, Massimo Vergassola, Bernard Dujon

**Affiliations:** 1 Unité de Génétique Moléculaire des Levures (CNRS URA 2171, UFR927 Université Pierre et Marie Curie), Département de Structure et Dynamique des Génomes, Institut Pasteur, Paris, France; 2 Unité Génétique des Génomes Bactériens (CNRS URA 2171), Département de Structure et Dynamique des Génomes, Institut Pasteur, Paris, France; 3 Atelier de Bioinformatique, Université Pierre et Marie Curie, Paris, France; 4 Laboratoire de Biologie Moléculaire de la Cellule (CNRS UMR 5161, INRA LA 1237), Ecole Normale Supérieure de Lyon, Lyon, France; 5 Unité de Génétique in silico (CNRS URA 2171), Département de Structure et Dynamique des Génomes, Institut Pasteur, Paris, France; Brandeis University, United States of America

## Abstract

Hemiascomycete yeasts cover an evolutionary span comparable to that of the entire phylum of chordates. Since this group currently contains the largest number of complete genome sequences it presents unique opportunities to understand the evolution of genome organization in eukaryotes. We inferred rates of genome instability on all branches of a phylogenetic tree for 11 species and calculated species-specific rates of genome rearrangements. We characterized all inversion events that occurred within synteny blocks between six representatives of the different lineages. We show that the rates of macro- and microrearrangements of gene order are correlated within individual lineages but are highly variable across different lineages. The most unstable genomes correspond to the pathogenic yeasts Candida albicans and Candida glabrata. Chromosomal maps have been intensively shuffled by numerous interchromosomal rearrangements, even between species that have retained a very high physical fraction of their genomes within small synteny blocks. Despite this intensive reshuffling of gene positions, essential genes, which cluster in low recombination regions in the genome of *Saccharomyces cerevisiae,* tend to remain syntenic during evolution. This work reveals that the high plasticity of eukaryotic genomes results from rearrangement rates that vary between lineages but also at different evolutionary times of a given lineage.

## Synopsis

The yeast Saccharomyces cerevisiae has proved to be a very powerful model organism for deciphering the molecular functioning of our cells. It also is the first eukaryote (the domain of life that includes human) whose genome has been completely sequenced in 1996. There are hundreds of species of yeast covering a tremendous genetic diversity. Almost ten years after the release of the first complete eukaryotic genome sequence, yeasts are still at the forefront of the field of genomics as they represent the monophyletic group of eukaryotes for which the largest number of complete genome sequences has been unveiled. The comparative analysis of their organization now provides an exquisite tool to dissect the mechanistic underpinnings of the process of genome evolution. This study reveals the extraordinary plasticity of the eukaryotic genomes. It also shows that genomes get rearranged at different rates both between the different lineages but also at the different evolutionary times of a given lineage. Finally, in spite of their distant phylogenetic relationship, pathogenic yeasts such as the two main causatives of human candidiasis, Candida albicans and Candida glabrata species, harbor the most unstable genomes of all lineages.

## Introduction

**Figure 1:**
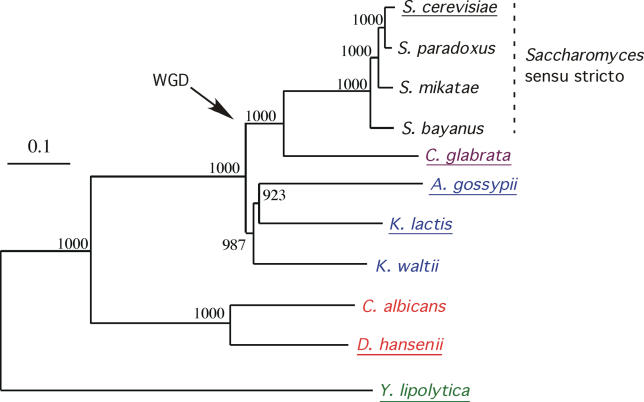
Phylogeny of Hemiascomycete Species The phylogenetic tree was built on the concatenated sequences of 25 proteins having clear orthologs in all of the 11 studied species. Bootstraps of the tree are given at the branches (out of 1,000). Species whose names are underlined correspond to fully sequenced genomes for which the number of supercontigs is identical to the number of chromosomes. The arrow indicates the place where the whole genome duplication (WGD) event occurred in the tree.

The class of Hemiascomycete comprises several hundreds of simple fungi, the vast majority of which are yeasts. Among them, there are few opportunistic pathogens such as Candida albicans that are responsible for the majority of all forms of candidiasis [1]. *Debaryomyces hansenii,* a cryotolerant species that tolerates high salinity levels, is a close relative to C. albicans (Figure 1). Although considered a nonpathogenic yeast, D. hansenii and its anamorph Candida famata have been associated with one case of bone infection and several cases of superficial infections [2,3]. The second causative agent of human candidiasis is *Candida glabrata.* In spite of its genera name, this species is phylogenetically more closely related to Saccharomyces cerevisiae than to C. albicans (Figure 1)*.* The level of genetic diversity between yeast species is often unsuspected. For instance, the average protein divergence of more than 50% found between S. cerevisiae and *Yarrowia lipolytica,* an alkane-using yeast, reveals that Hemiascomycetes are molecularly as diverse as the entire phylum of chordates [4]. The level of protein divergence within the *Saccharomyces* “sensu stricto” complex (see Figure 1), whose different members are thought to be in the early stages of the speciation process, already compares to the one found between mammals [4–7].

A high level of synteny conservation of more than 98% has been reported between the genomes of the *Saccharomyces* “sensu stricto” species [7–9]. The term synteny originally referred to gene loci that map on the same chromosome, but it is now commonly used to design chromosomal regions in different genomes that share a common evolutionary origin. In other words, two regions are named syntenic when multiple consecutive genes are found in a (nearly) conserved order between the two genomes considered. Homologous chromosomes between the *Saccharomyces* “sensu stricto” species are almost colinear, differing from each other by only few translocations and large inversions that cause macrosynteny breakpoints (i.e., the simultaneous relocalization or reorientation of many genes). It has also been shown that the rate of formation of translocations is not constant in this group of species, indicating that bursts of rearrangements might have occurred at some points of their evolutionary history [10]. In fact, the majority of gene order changes between these species corresponds to microsynteny breakpoints created by the alternative loss of duplicates in the different species [9]. A whole genome duplication event, that occurred after the divergence of the *Saccharomyces* and *Kluyveromyces* lineages [4,11–13] (see Figure 1), resulted in the sudden doubling of all gene copies. The return to the diploid state was accompanied by a massive loss of nearly 90% of the duplicated gene copies, leaving only one copy of each gene in each genome. The differential loss of the two copies in two different species has lead to microsynteny breakpoints between their genomes.

At broader evolutionary distances, the coincidence between chromosome maps is blurred by the accumulation of numerous interchromosomal rearrangements [4]. However, little is known about the degree and the rate of chromosomal reorganization in the different lineages. Nearly complete genome sequences are now available for numerous yeast species [4,7,12–17] so we assessed the influence of both macro- and microrearrangements onto the evolution of the genomic architectures of representative yeast species covering the entire Hemiascomycete phylum. In this study we used the complete (or nearly complete) genome sequences of 11 yeast species to quantify the rates of macrorearrangements by measuring the level of gene order conservation between pairs of species. We also identified all inversion events that occurred within synteny regions shared between the genomes of six fully sequenced species. We discovered a tremendous level of chromosomal reorganization outside of the *Saccharomyces* “sensu stricto” species and showed that different rates of both macro- and microrearrangements applied in the different yeast lineages.

## Results/Discussion

### Rates of Genome Instability

**Figure 2:**
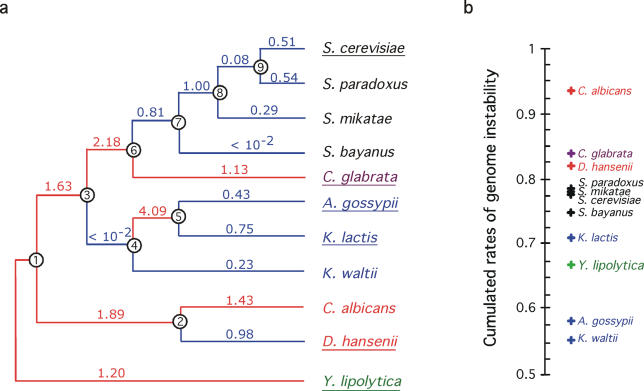
Rates of Genome Instability in Hemiascomycetes (A) Branch-specific normalized rates of genome rearrangements are indicated either in red or in blue illustrating higher or lower rates than average, respectively. Nodes are numbered from 1 to 9. (B) Cumulated rates of genome instability correspond to the ratios between the sum of the branch-specific GOL and the phylogenetic distance separating each species from node 1 (Table 2).

To quantify the relative rates of rearrangements in the different lineages we first computed a gene order conservation (GOC) index [18,19] between the 11 yeast species for which the phylogeny is presented in Figure 1. Putative orthologs were identified for all pairs of genomes and two pairs of orthologs are in a “relation of conserved order” if they are separated by less than four intervening genes in both genomes (Materials and Methods). GOC is the proportion of such syntenic pairs among the total number of orthologs between the two compared genomes. Hence, GOC varies between 0 (no pair of syntenic orthologs) and 1 (complete GOC). GOCs were calculated for the 55 pairs of species ([*n*(*n*−1)]/2, with *n* = 11) and a phylogenetic tree derived from the concatenated sequences of 25 orthologous proteins in the 11 genomes was constructed (see Materials and Methods) to estimate the evolutionary distances between all pairs of species (Figure 1). The tree topology was further supported by using the concatenated sequences of 16 ribosomal proteins to construct a second tree. The resulting topology is completely identical to that described in Figure 1 (not shown). Naturally, GOCs arising from the comparisons of closely related species are closer to one than the ones between distant species (Table 1). Reciprocally, the proportions of gene order loss (GOL = 1 − GOC) increase along with the phylogenetic distances (Table 1). We reasoned that each of the 55 interspecies GOL values results from the sum of all events of genome rearrangements that occurred in the different branches separating two species from their last common ancestor on the phylogenetic tree. There are 19 branches in total on the phylogenetic tree for which branch-specific GOL can be estimated. We inferred them from the 55 interspecies GOL values presented in Table 1. Branch-specific GOLs were calculated by minimizing the sum, over the 55 pairwise comparisons, of the squared differences between the frequency of observed genome rearrangements (GOL) and the sum of the predicted branch-specific GOL values (Materials and Methods). The resulting branch-specific GOL values are presented in Table 2. We checked that the sum of the branch-specific GOL values between two species gave an estimation (GOL_est_) close to the original GOL values obtained from the GOC analysis (Table 1). For instance, GOL_est_ between Y. lipolytica and D. hansenii (Table 1) is the sum of the three branch-specific GOL values *x_1_, x_2_,* and *x_3_* (Table 2). It differs from the original GOL value between Y. lipolytica and D. hansenii by 1.4% only (Table 1). For all 55 pairwise comparisons, differences between GOL_est_ and the original GOL values are very limited (average 3.2%, min = 0%, max = 11.6%, Table 1). Branch-specific rates of genome rearrangements were obtained by dividing the branch-specific GOL values by the length of their corresponding branches in the tree and centered around one by dividing them by the mean rate (Table 2). Normalized GOL rates for the 19 branches are presented on Figure 2A with a color code indicating rates of gene order rearrangements either higher (red) or lower (blue) than average. It clearly appears that rates of rearrangements remained high in all branches from node 1 to the two main causative agents of human candidiasis, C. albicans and *C. glabrata.* Given the external position of Y. lipolytica on the phylogenetic tree, only one branch covers the entire lineage from node 1 to the present-day species. Globally, deep branches of the tree that stem out from node 1 present high rearrangement rates. A general decrease of the rates is observed both in the *Saccharomyces* and in the *Kluyveromyces/Ashbya* lineages. Rearrangements have also slowed down in the D. hansenii–specific branch since it diverged from *C. albicans.* In addition, the concomitant presence of both stable (in the *Saccharomyces* species) and unstable (in C. glabrata) branches subsequent to the ancestral genome duplication suggests that rates of rearrangements have not been influenced by this event. Except for the external species, *Y. lipolytica,* rates of rearrangements can be compared either between species-specific terminal branches only, or globally over the whole evolutionary distance between node 1 and the present-day species. Rates of rearrangements on terminal branches give an estimate of the most recent level of genome instability. The most stable genome corresponds to that of S. bayanus followed by those of K. waltii and *S. mikatae,* while the most unstable ones correspond to those from the pathogenic yeasts, C. albicans and C. glabrata (Figure 2A). These two species also present the highest cumulated rates of genome instability when entire evolutionary distances since node 1 are taken into account (Figure 2B). C. albicans and C. glabrata yeasts occupy narrow ecological niches and in the process of becoming more specialized, their genomes may have accumulated more rearrangements because of selective sweeps or because of lower population sizes leading to less efficient selection onto gene order. It is also possible that the population structures of these pathogenic yeasts that are largely if not entirely clonal due to the lack of mating might contribute to the apparent genome plasticity. At the other end of the scale, the most stable genomes during evolution correspond to the *Kluyveromyces/Ashbya* lineage as well as that of *Y. lipolytica.*

**Table 1:**
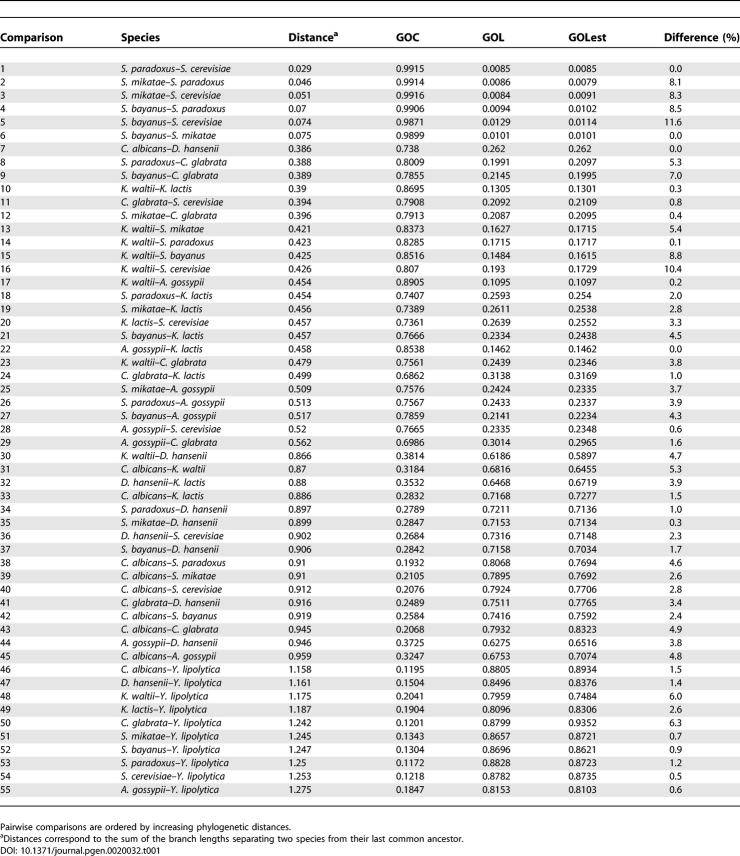
GOC and GOL between the 55 Pairwise Comparisons

**Table 2:**
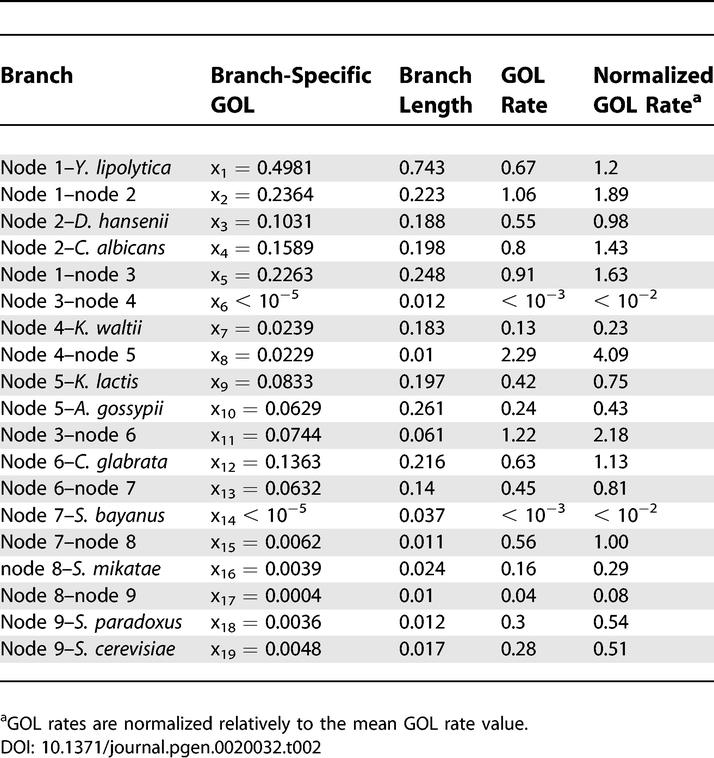
Branch-Specific Rates of GOL

### Small Inversions within Synteny Blocks

**Figure 3:**
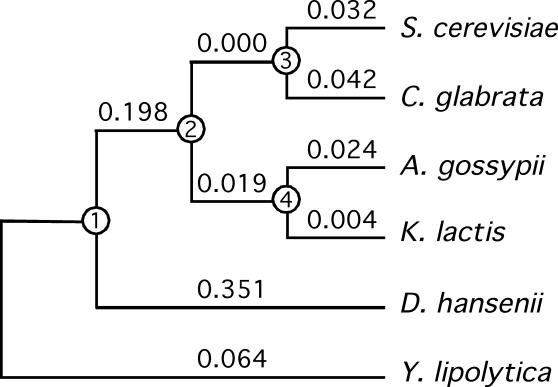
Branch-Specific Expected Number of Inversions per Gene The tree topology is deduced from Figure 1. The nodes are numbered from 1 to 4. Calculated inversion numbers are indicated on each branch of the tree.

Variable rates of microrearrangements of gene order were also found between the different lineages. Microrearrangements were sought by characterizing all small inversions that occurred within the synteny blocks (Materials and Methods) shared between the six fully sequenced yeast genomes [4,12,14] (underlined species on Figure 1). The total number of inversions between two genomes varies by more than one order of magnitude depending on the species (Table 3). The mean number of inversions per synteny block as well as the mean frequency of gene inversion show that in spite of the largest synteny blocks, A. gossypii and K. lactis have undergone much fewer inversions than any other couple of species, even fewer than S. cerevisiae and *C. glabrata,* which are more closely related. Inversions are found in only 10% of the synteny blocks between A. gossypii and *K. lactis,* while there is on average more than one inversion per synteny block between D. hansenii and any of the other species. In all comparisons involving *D. hansenii,* the mean frequencies of gene inversion range between 0.42 and 0.65 (i.e., approximately half of the genes have been inverted). Despite a much larger evolutionary distance, the mean frequencies of gene inversions are twice as small for comparisons involving Y. lipolytica. Branch-specific expected numbers of inversion per gene were extracted from these pairwise comparisons by minimizing the sum of the relative errors (see Materials and Methods). The D. hansenii branch shows, by far, the highest rate of gene inversion (0.351, Figure 3). By comparison, a very low inversion rate applies in the Y. lipolytica branch (0.064). A global decrease in the inversion rates occurred in all branches leading to the four other species from their last common ancestor (i.e., from node 2 on Figure 3) and this trend is even more pronounced in the A. gossypii– and K. lactis–specific branches (originating from node 4) than in the S. cerevisiae– and C. glabrata–specific branches (originating from node 3). Note that the null value of the branch between nodes 2 and 3 is due to the fact that the numbers of gene inversions in pairwise comparisons involving S. cerevisiae are very close to the numbers of inversions in the corresponding pairwise comparisons involving C. glabrata (see Table 3). These variable rates of microrearrangements of gene order are fully consistent with the relative rates of macrorearrangements characterized above (Figure 2). Previous works based on partial sequences of yeast genomes pointed out that the proportions of locally inverted genes remained rare over a relatively long evolutionary distance (less than 5% between the *Saccharomyces* and the Kluyveromyces spp.), [8] but become prominent over longer evolutionary distances (30% to 40% between *S. cerevisiae* and C. albicans or D. hansenii) [8,20]. Here we show that this difference is not solely due to the phylogenetic distances but relies on an accelerated rate of rearrangement in the *C. albicans/D. hansenii* lineage as compared to much slower rates in the *Kluyveromyces* and *Saccharomyces* groups of species (Figure 3).

**Table 3:**
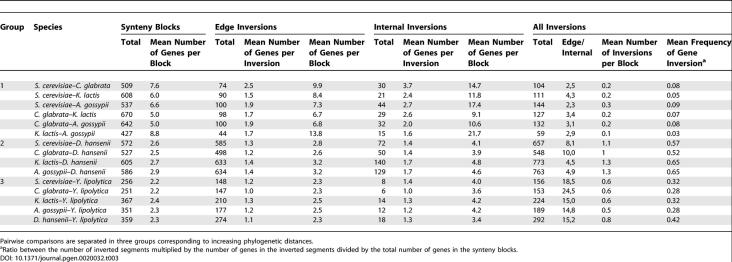
Small Inversions within Synteny Blocks between Six Representative Species of Hemiascomycetes

Inversions are categorized as “internal” when the whole inverted segment is comprised within a synteny block or as “edge” when one end of the inverted segment coincides with the end of the block. In all 15 pairwise comparisons, edge inversions are far more frequent than internal ones and the ratio between these two categories increases with the phylogenetic distances (Table 3). In addition, the length of the synteny blocks that contain edge inversions is on average smaller than the length of blocks carrying internal inversions only. These observations suggest that edge inversions could correspond in fact to internal inversion events that were subsequently interrupted by a synteny breakpoint. Indeed, synteny breakpoints occurring within an inverted segment would not only produce two new edge inversions but would also result in a decrease of the size of the two resulting synteny blocks. The small size of the edge-containing blocks of synteny as well as the increasing proportions of edge inversions at larger phylogenetic distances are fully compatible with such a scenario of formation of edge inversions. This also implies that the original events of inversion were probably larger than the size of the remaining inverted segments at the edge of the synteny blocks. The average length of the original events of inversion was estimated from the sole internal events. It varies from one gene, for comparisons between distant species, to 3.7 genes between the two closest species (S. cerevisiae versus *C glabrata,* Table 3). Despite a possible underestimation of the inversion sizes within the *S. cerevisiae/C. glabrata* group (due to the relatively small size of the synteny blocks), inversions appear to be significantly longer between these two species than between K. lactis and A. gossypii (mean length of 3.7 ± 0.5 genes versus 1.6 ± 0.4 genes, respectively).

### Reorganization of the Chromosomal Maps

**Figure 4:**
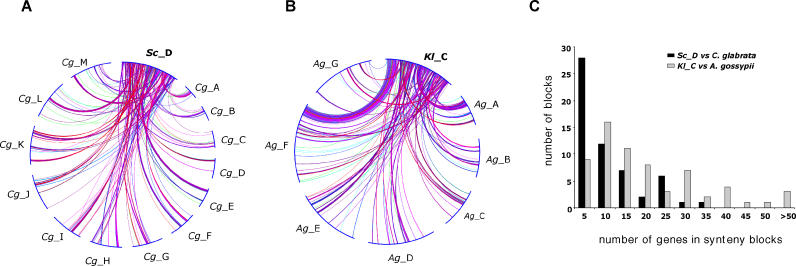
Chromosomal Map Reorganization between Related Species The circular representation is adapted from [25]. (A) Chromosome D from S. cerevisiae (*Sc*_D) is represented in a circle with the 13 chromosomes from C. glabrata (*Cg*_A to M). Each line joins two orthologs and the color of the lines represents the percentage of similarity between orthologous gene products (green ≤ 50% ≤ cyan ≤ 60% ≤ blue ≤ 70% ≤ magenta ≤ 80% ≤ dark magenta ≤ 90% ≤ red). (B) Same representation between chromosome C of K. lactis (*Kl*_C) and the seven chromosomes from A. gossypii (*Ag*_A to G). The diagram shows large uninterrupted regions of conserved synteny between *Kl_*C and *Ag*_F or *Ag*_A, while no such conservation is visible between *Sc*_D and any of the C. glabrata chromosomes. (C) Distribution of the length of the corresponding syntenic blocks between *Sc*_D and C. glabrata (black) and *Kl_*C and A. gossypii (gray).

The higher genomic stability in the *K. lactis*/A. gossypii lineage as compared to the S. cerevisiae/C. glabrata group of species is directly observable at the chromosomal level. In spite of its genera name, *C. glabrata* is the closest relative to the *Saccharomyces* clade with a fully sequenced genome. A slightly larger phylogenetic distance separates K. lactis from A. gossypii than S. cerevisiae from C. glabrata. However, chromosomal colinearity is better preserved between the former pair. Large uninterrupted chromosomal regions are still recognizable between some of the K. lactis and A. gossypii chromosomes, while any individual chromosome of S. cerevisiae is scattered into small and intermingled pieces onto virtually all of the chromosomes of *C. glabrata* (Figures 4, S1, and S2). By contrast, very few macrorearrangements have disrupted the chromosomal colinearity between the genomes of the *Saccharomyces* “sensu stricto” species [7,10]. This underlines an important evolutionary leap in the level of chromosomal reorganization between the “sensu stricto” species and *C. glabrata.* Interestingly, in spite of the important level of chromosome map reshuffling, a very high fraction of the genomes of S. cerevisiae and C. glabrata are conserved within synteny blocks. The total length spanned by the synteny blocks between S. cerevisiae and C. glabrata represents 88% of the physical length of these genomes. This proportion rises to 93% when subtelomeric regions are excluded from the analysis, as no conservation of synteny was found between these regions. Although almost the entire genomes of these species are comprised within small synteny blocks, the global chromosomal colinearity has been completely destroyed by the accumulation of numerous and overlapping interchromosomal rearrangements. This clearly shows that loss of synteny primarily results from the accumulation of chromosomal rearrangements rather than from sequence divergence between orthologous regions that would impede recognition of their common evolutionary origin. It is also notable that, consistent with a higher level of chromosomal reorganization in the *S. cerevisiae/C. glabrata* than in the *K. lactis/A. gossypii* lineages, the size of the syntenic blocks is on average smaller in the former than in the latter (Table 3, Figure S3). The smaller size of the synteny blocks in *S. cerevisiae/C. glabrata* is also attributable to the massive gene loss that occurred subsequent to the whole genome duplication event [11], whereas the corresponding regions in the K. lactis genome have retained virtually all genes.

### Constraints on Gene Order Changes

**Figure 5:**
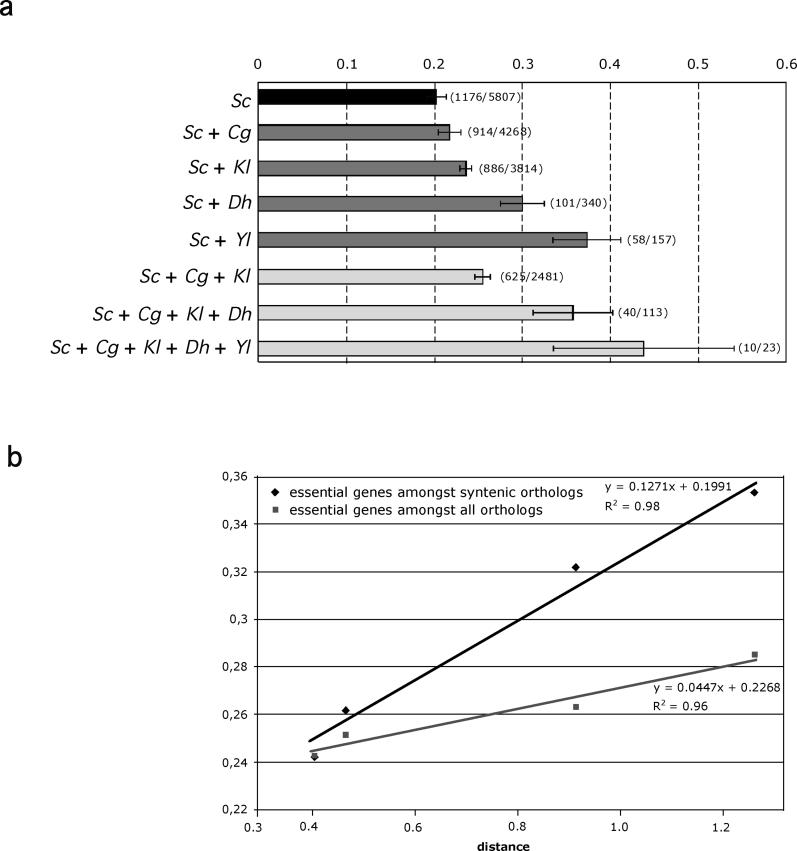
Proportion of Essential Genes within Conserved Synteny Blocks (A) The black bar represents the proportion of essential genes in the genome of *S. cerevisiae (Sc),* as defined in the Comprehensive Yeast Genome Database (http://mips.gsf.de/genre/proj/yeast). The relative proportions of orthologs to these genes among the total number of genes comprised within the syntenic blocks with the genomes of *C. glabrata (Cg), K. lactis (Kl), D. hansenii (Dh),* and *Y. lipolytica (Yl)* are represented by dark gray bars. Proportions of essential genes concomitantly conserved within synteny blocks in three, four, and five species are indicated by light gray bars. Error bars represent two standard deviations, and the number of genes considered in each case is indicated in parentheses. (B) Comparison of the proportions of essential genes among syntenic orthologs and among all orthologs at increasing phylogenetic distances. Phylogenetic distances between S. cerevisiae and *C. glabrata, S. cerevisiae* and *K. lactis, S. cerevisiae* and *D. hansenii,* and *S. cerevisiae* and *Y. lipolytica* are reported on the *x*-axis.

Genome dynamics results from the accumulation of both micro- and macrorearrangements and leads to an apparent randomization of gene order between distantly related yeast species. However, there is some evidence that gene order is under selection in eukaryotes [21]. In *S. cerevisiae,* essential genes tend to be clustered and these clusters are in regions of low recombination rates [22]. If gene order is constrained by natural selection, synteny breaks within such clusters would be counter-selected. We determined the proportions of genes in synteny blocks that are essential (i.e., those for which the knockout is lethal in S. cerevisiae) between representative species of the different lineages. This proportion increases with the phylogenetic distance between species (Figure 5A). This trend is even stronger for essential genes concomitantly conserved in synteny between three, four, or the five compared species. This suggests that essential genes tend to remain clustered within genomes during evolution. However, essential genes evolve more slowly than nonessential ones [23]. Therefore, this increase could be due, at least partly, to the better sequence conservation of essential genes that would result in a higher proportion of such genes among all identified orthologs. We plotted the proportion of essential genes among all orthologs for the four pairwise comparisons and showed that it increases along with the phylogenetic distance but to a significantly lower rate than the proportion of essential genes in synteny (Figure 5B). Altogether, these results show that essential genes tend to remain adjacent during evolution, and this trend remains observable even at very large evolutionary distances where genomes have been massively reshuffled by chromosomal rearrangements.

### Future Prospects

This work shows that genome dynamics varies very significantly between related yeast species. However, within each genome macro- and microrearrangements occur at similar relative rates. In higher eukaryotes, a slow rate of genome reorganization has been characterized in human compared to that of rodent, and an even slower rate has been characterized in chicken [24]. A very slow rate of interchromosomal rearrangements has also been described for the very compact genome of *Tetraodon* [25]. Rates of chromosome evolution have also been compared between eight mammalian species [32]. In addition to variations between the different orders, the authors characterized a global increase in breakage rates after the Cretaceous-Tertiary boundary. These results are fully consistent with our findings that rearrangement rates not only vary between different yeast lineages but also at different evolutionary times of a given lineage. It remains to be understood why such differences exist. One could invoke intrinsic reasons to explain why some genomes can be less stable than others (e.g., because they could contain a higher proportion of repetitive sequences [transposable elements, duplicated genes] and/or because DNA damages would be less efficiently repaired). Moreover, selection is likely to act differently in different genomes. In this case, rearrangements could be fixed more frequently in yeasts with smaller effective population sizes, as it is probably the case for the pathogenic ones.

## Materials and Methods

### Orthology searches and GOC.

Genes were regarded as putative orthologs in pairwise comparisons if their products were reciprocal best-hits with at least 40% similarity in sequence and their sequences were less than 30% different in length, as in [18]. For the genomes of *S. mikatae, S. paradoxus, S. bayanus,* and *K. waltii,* where the annotations were not available, we mapped the genes within the contigs using FASTA searches [26]. We retained only the hits that were the best matches both in terms of score and E-value (and this smaller than E-10). Genome sequences were downloaded from http://www.yeastgenome.org for *S. cerevisiae,* http://cbi.labri.fr/Genolevures/index.php for *C. glabrata, K. lactis, D. hansenii,* and *Y. lipolytica,* http://agd.unibas.ch for *A. gossypii,* http://www.broad.mit.edu/seq/YeastDuplication for *K. waltii,* http://www.genome.wustl.edu/projects/yeast for *S. paradoxus, S. mikatae,* and *S. bayanus,* and http://www.candidagenome.org for *C. albicans.*

The GOC index was adapted from previous works [18,19] by allowing the presence of intervening genes between syntenic pairs of orthologs in order to recover most relations of GOC that would otherwise be lost due to the massive gene loss that occurred after the whole genome duplication event. After some experimentation, the upper limit was set to four intervening genes, as larger neighborhoods typically led to a GOC less than 1% higher but a smaller statistical confidence. Synteny blocks were defined as series of neighboring syntenic pairs of orthologs separated by less than ten intervening genes in both compared genomes.

### Phylogenetic analysis.

The distance matrix between the species was computed using maximum likelihood with Tree-Puzzle [27] (JTT + Γ(8) model). We built two phylogenetic trees, one using 25 randomly chosen highly conserved ubiquitous genes (orthologs to *YAL044w-a, YAL016w, YBL057c, YBR025c, YBR127c, YCR009c, YCR011c, YDL031w, YDR140w, YDR449c, YER068w, YER110c, YER141w, YGR096w, YGR235c, YIL043c, YJR010w, YJR096w, YKL184w, YKL134c, YKL103c, YLR351c, YNL062c, YNR007c,* and *YPR051w*), and another using 16 ubiquitous ribosomal proteins. Both trees had exactly the same topology. Trees were built from the distance matrix using BIONJ [27] and the robustness of the branches was assessed with 1,000 bootstraps using BOOTSEQ and CONSENSE from the PHYLIP package [28].

### Inversions.

We searched for local inversions within synteny blocks using the DERANGE algorithm [29]. This program is intended to find the most economical number of moves (inversions, transpositions, and transversions) to transform an ordered and orientated sequence of *n* genes in the first genome to the actual order of the corresponding *n* orthologs in the second genome. When all types of move, inversions (e.g., a sequence of four genes, A B C D, becomes A –B C D, with “–” denoting a switch of coding strand for gene B), transpositions (e.g., A B C D becomes A C D B), or transversions (e.g., A B C D becomes A C D –B) are assigned the same weight, inversions appear to be far more frequent than transpositions or transversions (65% to 85% of the moves). Transversions and transpositions were massively penalized as all gene order/orientation changes observed within synteny blocks can easily be explained by the only mean of inversions, even if this tends to increase the total number of moves (from 10% to 25% depending on the compared species). The few remaining synteny blocks still containing transposition or transversion events were analyzed with the GRIMM-Synteny program [30] to reconstruct, by inversions only, the gene order/orientation changes that occurred in the corresponding regions.

### Calculation of branch-specific values.

Branch-specific GOL values, *x_j_,* were calculated by minimizing the following equation

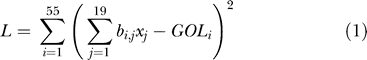
where *b_i,j_* is a Boolean variable indicating if the branch-specific GOL variable *x_j_* (Table 2) contributes to the *i*-th interspecies comparison and GOL_i_ are the values measured in the interspecies comparisons (GOL_i_ = 1 − GOC_i_). For example, in the 47th comparison between Y. lipolytica and D. hansenii (Table 1), *b*_47,1_ *= b*_47,2_ *= b*_47,3_ *= 1* and all the others are zero. The resulting optimization problem is quadratic, with the constraints that all variables *x_j_* must be positive. It is easy to verify the convexity of the quadratic form *L* to be minimized (positive Hessian), ensuring the uniqueness of the minimum, which is computed solving the linear Karush-Kuhn-Tucker optimality conditions by matrix inversion [31].

Expected numbers of inversions per gene on each of the nine branches of the phylogenetic tree on Figure 3 were inferred likewise by minimizing the sum, over the 15 pairwise comparisons, of the squared differences between the number of predicted inversions and the number of inversions observed in the pairwise comparisons. For each pair of species, the former is given by the sum of the expected number of inversions per gene along the branches separating the two species.

## Supporting Information

Figure S1 Chromosomal Map Reorganization between S. cerevisiae and C. glabrata
Each chromosome from S. cerevisiae (SACE_A to P) is represented in a circle with the 13 chromosomes from C. glabrata (CAGL_A to M). Each line joins two orthologs and the color of the lines represents the percentage of similarity between orthologous gene products (green ≤ 50% ≤ cyan ≤ 60% ≤ blue ≤ 70% ≤ magenta ≤ 80% ≤ dark magenta ≤ 90% ≤ red).
(1.1 MB PDF)

Figure S2 Chromosomal Map Reorganization between K. lactis and A. gossypii
Each chromosome from K. lactis (KLLA_A to F) is represented in a circle with the seven chromosomes from A. gossypii (ASGO_A to G). Each line joins two orthologs and the color of the lines represents the percentage of similarity between orthologous gene products (green ≤ 50% ≤ cyan ≤ 60% ≤ blue ≤ 70% ≤ magenta ≤ 80% ≤ dark magenta ≤ 90% ≤ red).
(630 KB PDF)

Figure S3 Distribution of the Length of the Syntenic Blocks between S. cerevisiae and C. glabrata (Black Bars) and between K. lactis and A. gossypi (Gray Bars) (23 KB PDF)

## Abbreviations

GOC: gene order conservation
GOL: gene order loss

## Note Added in Proof

Reference 32 was added while this paper was in proofs stage; as a result, it is cited out of order in the text.

## References

[pgen-0020032-b001] Pfaller MA, Jones RN, Doern GV, Sader HS, Messer SA (2000). Bloodstream infections due to *Candida* species: SENTRY antimicrobial surveillance program in North America and Latin America, 1997–1998. Antimicrob Agents Chemother.

[pgen-0020032-b002] Nishikawa A, Tomomatsu H, Sugita T, Ikeda R, Shinoda T (1996). Taxonomic position of clinical isolates of Candida famata. J Med Vet Mycol.

[pgen-0020032-b003] Wong B, Kiehn TE, Edwards F, Bernard EM, Marcove RC (1982). Bone infection caused by Debaryomyces hansenii in a normal host: A case report. J Clin Microbiol.

[pgen-0020032-b004] Dujon B, Sherman D, Fischer G, Durrens P, Casaregola S (2004). Genome evolution in yeasts. Nature.

[pgen-0020032-b005] Makalowski W, Boguski MS (1998). Evolutionary parameters of the transcribed mammalian genome: An analysis of 2,820 orthologous rodent and human sequences. Proc Natl Acad Sci U S A.

[pgen-0020032-b006] Malpertuy A, Tekaia F, Casaregola S, Aigle M, Artiguenave F (2000). Genomic exploration of the hemiascomycetous yeasts: 19. Ascomycetes—Specific genes. FEBS Lett.

[pgen-0020032-b007] Kellis M, Patterson N, Endrizzi M, Birren B, Lander ES (2003). Sequencing and comparison of yeast species to identify genes and regulatory elements. Nature.

[pgen-0020032-b008] Llorente B, Malpertuy A, Neuveglise C, de Montigny J, Aigle M (2000). Genomic exploration of the hemiascomycetous yeasts: 18. Comparative analysis of chromosome maps and synteny with Saccharomyces cerevisiae. FEBS Lett.

[pgen-0020032-b009] Fischer G, Neuveglise C, Durrens P, Gaillardin C, Dujon B (2001). Evolution of gene order in the genomes of two related yeast species. Genome Res.

[pgen-0020032-b010] Fischer G, James SA, Roberts IN, Oliver SG, Louis EJ (2000). Chromosomal evolution in *Saccharomyces*. Nature.

[pgen-0020032-b011] Wolfe KH, Shields DC (1997). Molecular evidence for an ancient duplication of the entire yeast genome. Nature.

[pgen-0020032-b012] Dietrich FS, Voegeli S, Brachat S, Lerch A, Gates K (2004). The Ashbya gossypii genome as a tool for mapping the ancient Saccharomyces cerevisiae genome. Science.

[pgen-0020032-b013] Kellis M, Birren BW, Lander ES (2004). Proof and evolutionary analysis of ancient genome duplication in the yeast Saccharomyces cerevisiae. Nature.

[pgen-0020032-b014] Goffeau A, Barrell BG, Bussey H, Davis RW, Dujon B (1996). Life with 6000 genes. Science.

[pgen-0020032-b015] Jones T, Federspiel NA, Chibana H, Dungan J, Kalman S (2004). The diploid genome sequence of Candida albicans. Proc Natl Acad Sci U S A.

[pgen-0020032-b016] Cliften P, Sudarsanam P, Desikan A, Fulton L, Fulton B (2003). Finding functional features in *Saccharomyces* genomes by phylogenetic footprinting. Science.

[pgen-0020032-b017] Liti G, Louis EJ (2005). Yeast evolution and comparative genomics. Annu Rev Microbiol.

[pgen-0020032-b018] Rocha EP (2003). DNA repeats lead to the accelerated loss of gene order in bacteria. Trends Genet.

[pgen-0020032-b019] Rocha EP (2005). Inference and analysis of the relative stability of bacterial chromosomes. Mol Biol Evol.

[pgen-0020032-b020] Seoighe C, Federspiel N, Jones T, Hansen N, Bivolarovic V (2000). Prevalence of small inversions in yeast gene order evolution. Proc Natl Acad Sci U S A.

[pgen-0020032-b021] Hurst LD, Pal C, Lercher MJ (2004). The evolutionary dynamics of eukaryotic gene order. Nat Rev Genet.

[pgen-0020032-b022] Pal C, Hurst LD (2003). Evidence for co-evolution of gene order and recombination rate. Nat Genet.

[pgen-0020032-b023] Wall DP, Hirsh AE, Fraser HB, Kumm J, Giaever G (2005). Functional genomic analysis of the rates of protein evolution. Proc Natl Acad Sci U S A.

[pgen-0020032-b024] Bourque G, Zdobnov EM, Bork P, Pevzner PA, Tesler G (2005). Comparative architectures of mammalian and chicken genomes reveal highly variable rates of genomic rearrangements across different lineages. Genome Res.

[pgen-0020032-b025] Jaillon O, Aury JM, Brunet F, Petit JL, Stange-Thomann N (2004). Genome duplication in the teleost fish Tetraodon nigroviridis reveals the early vertebrate proto-karyotype. Nature.

[pgen-0020032-b026] Pearson WR (1990). Rapid and sensitive sequence comparison with FASTP and FASTA. Methods Enzymol.

[pgen-0020032-b027] Schmidt HA, Strimmer K, Vingron M, von Haeseler A (2002). TREE-PUZZLE: Maximum likelihood phylogenetic analysis using quartets and parallel computing. Bioinformatics.

[pgen-0020032-b028] Felsenstein J (2005). PHYLIP (Phylogeny Inference Package) version 3.6 [computer program]. Distributed by the author.

[pgen-0020032-b029] Blanchette M, Kunisawa T, Sankoff D (1996). Parametric genome rearrangement. Gene.

[pgen-0020032-b030] Pevzner P, Tesler G (2003). Genome rearrangements in mammalian evolution: Lessons from human and mouse genomes. Genome Res.

[pgen-0020032-b031] Bazaraa M, Sherali D, Shetty C (1992). Nonlinear programming: Theory and algorithms.

[pgen-0020032-b032] Murphy WJ, Larkin DM, Everts-van der Wind A, Bourque G, Tesler G (2005). Dynamics of mammalian chromosome evolution inferred from multispecies comparative maps. Science.

