# Supplementary material for: Highly Variable Rates of Genome Rearrangements between Hemiascomycetous Yeast Lineages
Source: PLoS Genet. 2006 Mar 10;2(3):e32. doi: 10.1371/journal.pgen.0020032 (PMC1391921; doi:10.1371/journal.pgen.0020032)
Supplement: Supplementary file 1 [file pgen.0020032.sg001.pdf]

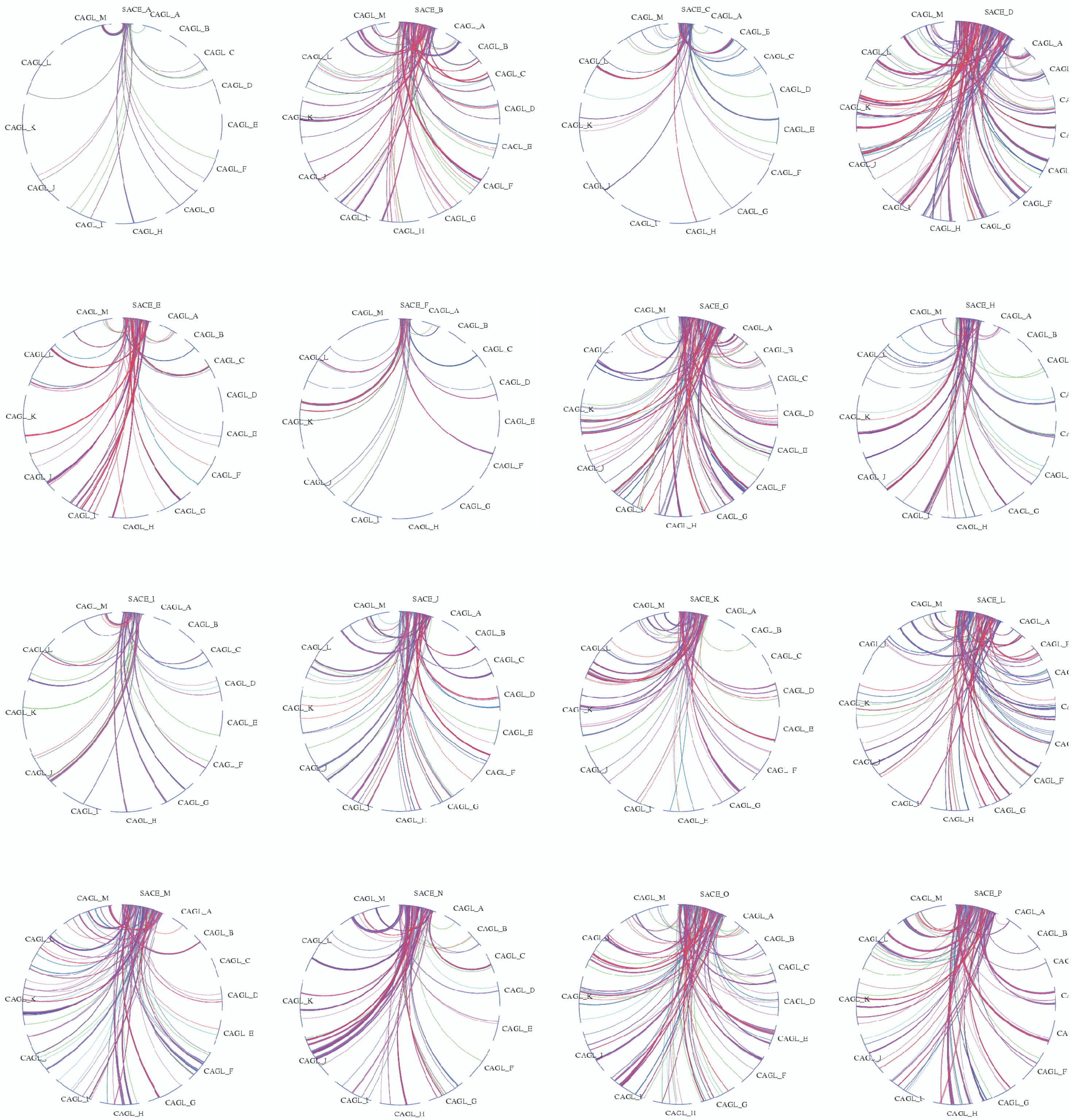
SACE_A
SACE_B
SACE_C
SACE_D
SACE_E
SACE_F
SACE_G
SACE_H
SACE_I
SACE_J
SACE_K
SACE_L
SACE_M
SACE_N
SACE_O
SACE_P
CAGL_A
CAGL_B
CAGL_C
CAGL_D
CAGL_E
CAGL_F
CAGL_G
CAGL_H
CAGL_I
CAGL_J
CAGL_K
CAGL_L
CAGL_M
