# Supplementary material for: Highly Variable Rates of Genome Rearrangements between Hemiascomycetous Yeast Lineages
Source: PLoS Genet. 2006 Mar 10;2(3):e32. doi: 10.1371/journal.pgen.0020032 (PMC1391921; doi:10.1371/journal.pgen.0020032)
Supplement: Supplementary file 2 [file pgen.0020032.sg002.pdf]

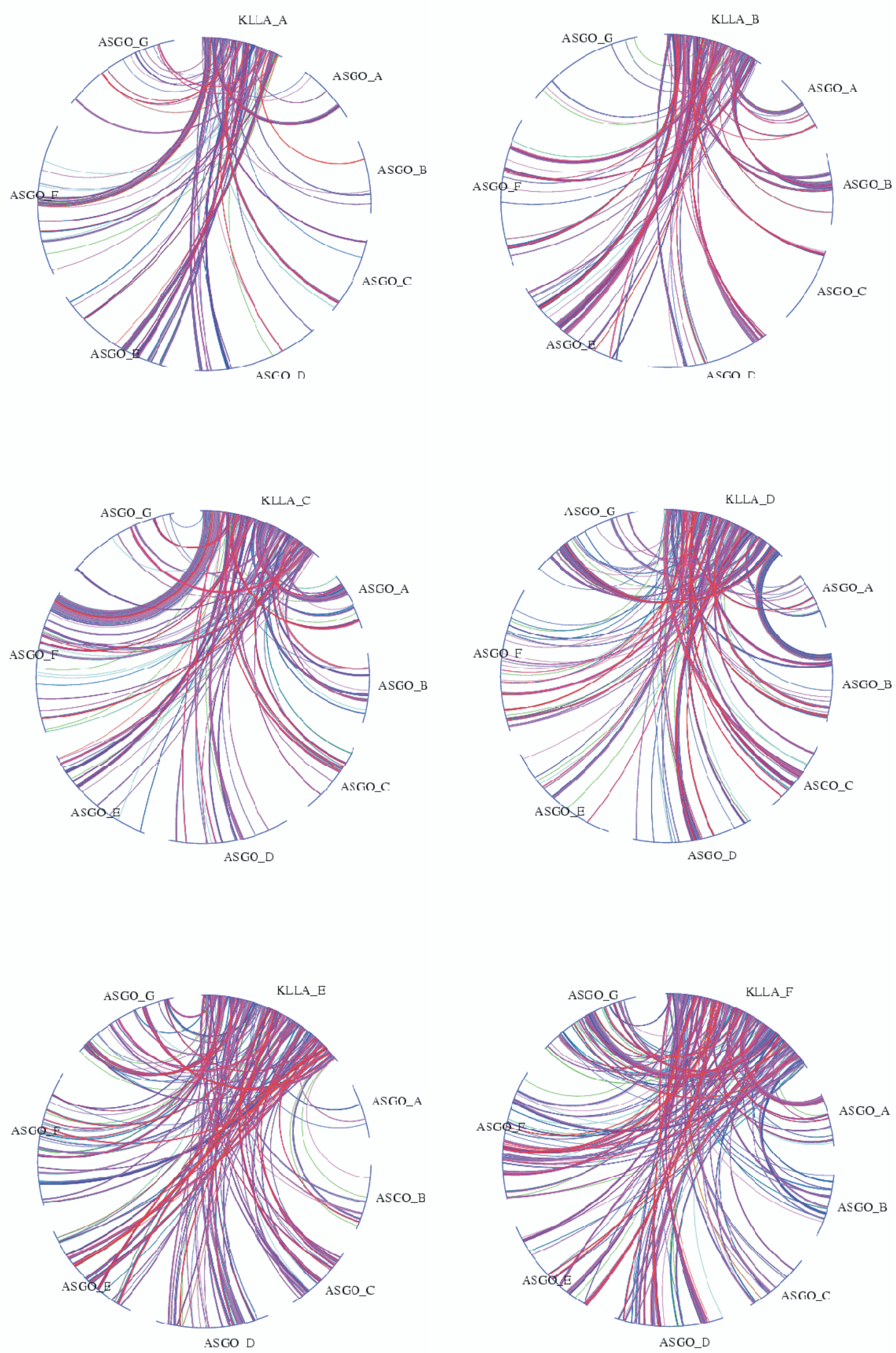
KLLA_A
ASGO_G
ASGO_A
ASGO_B
ASGO_F
ASGO_C
ASGO_E
ASGO_D
KLLA_B
ASGO_G
ASGO_A
ASGO_B
ASGO_F
ASGO_C
ASGO_E
ASGO_D
KLLA_C
ASGO_G
ASGO_A
ASGO_F
ASGO_B
ASGO_C
ASGO_E
ASGO_D
KLLA_D
ASGO_G
ASGO_A
ASGO_F
ASGO_B
ASGO_C
ASGO_E
ASGO_D
KLLA_E
ASGO_G
ASGO_A
ASGO_F
ASGO_B
ASGO_E
ASGO_C
ASGO_D
KLLA_F
ASGO_G
ASGO_A
ASGO_F
ASGO_B
ASGO_E
ASGO_C
ASGO_D
