# Supplementary material for: Highly Variable Rates of Genome Rearrangements between Hemiascomycetous Yeast Lineages
Source: PLoS Genet. 2006 Mar 10;2(3):e32. doi: 10.1371/journal.pgen.0020032 (PMC1391921; doi:10.1371/journal.pgen.0020032)
Supplement: Supplementary file 3 [file pgen.0020032.sg003.pdf]

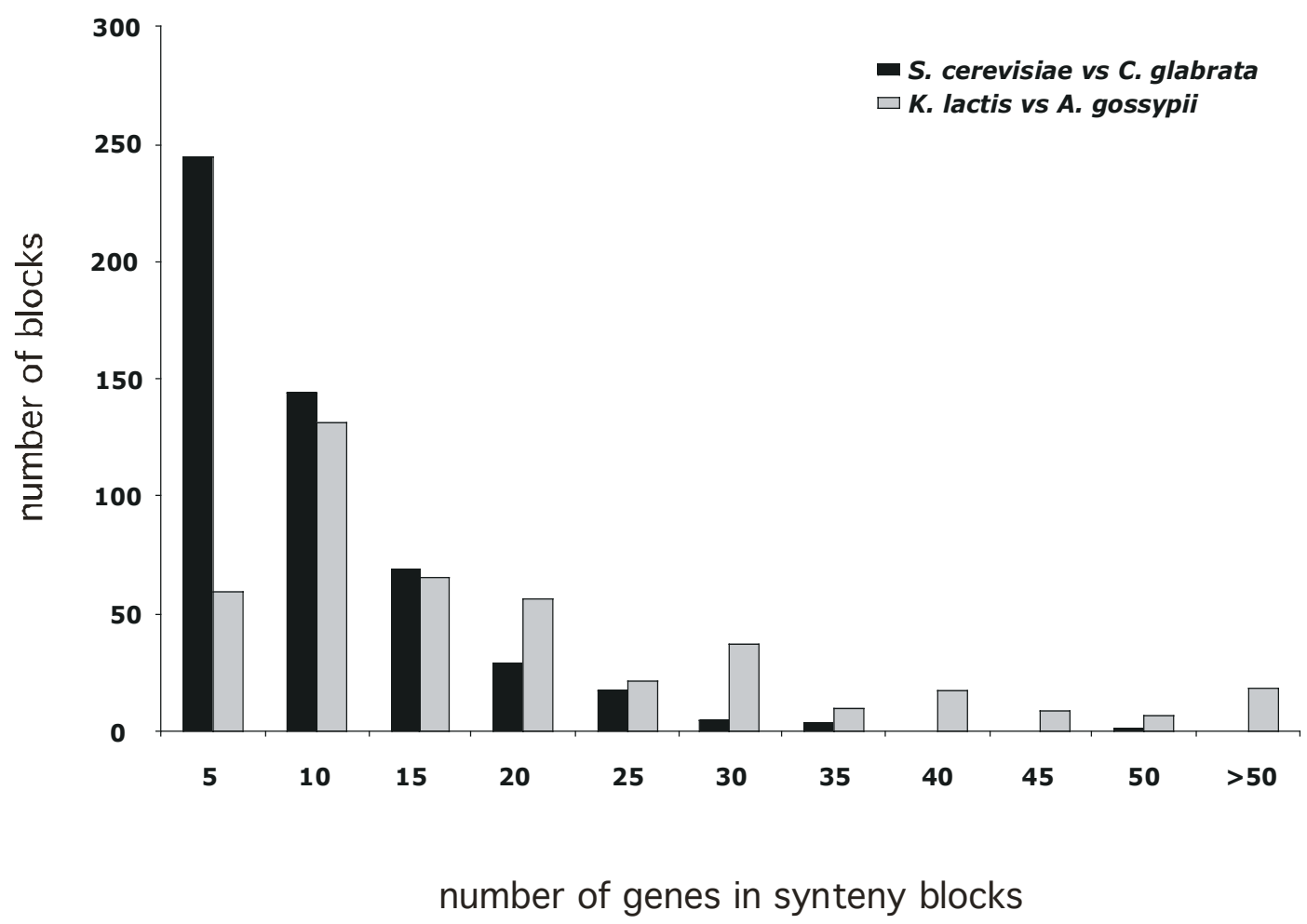

S. cerevisiae vs C. glabrata
K. lactis vs A. gossypii
300
250
200
150
100
50
0
number of blocks
5
10
15
20
25
30
35
40
45
50
>50
number of genes in synteny blocks
